# Relation between Belief and Performance in Perceptual Decision Making

**DOI:** 10.1371/journal.pone.0096511

**Published:** 2014-05-09

**Authors:** Jan Drugowitsch, Rubén Moreno-Bote, Alexandre Pouget

**Affiliations:** 1 Department of Brain and Cognitive Sciences, University of Rochester, Rochester, New York, United States of America; 2 Institut National de la Santé et de la Recherche Médicale, École Normale Supérieure, Paris, France; 3 Département des Neurosciences Fondamentales, Université de Genève, Geneva, Switzerland; 4 Research Unit, Parc Sanitari Sant Joan de Déu and Universitat de Barcelona, Barcelona, Spain; 5 Centro de Investigación Biomédica en Red de Salud Mental (CIBERSAM), Barcelona, Spain; Centre de Neuroscience Cognitive, France

## Abstract

In an uncertain and ambiguous world, effective decision making requires that subjects form and maintain a belief about the correctness of their choices, a process called meta-cognition. Prediction of future outcomes and self-monitoring are only effective if belief closely matches behavioral performance. Equality between belief and performance is also critical for experimentalists to gain insight into the subjects' belief by simply measuring their performance. Assuming that the decision maker holds the correct model of the world, one might indeed expect that belief and performance should go hand in hand. Unfortunately, we show here that this is rarely the case when performance is defined as the percentage of correct responses for a fixed stimulus, a standard definition in psychophysics. In this case, belief equals performance only for a very narrow family of tasks, whereas in others they will only be very weakly correlated. As we will see it is possible to restore this equality in specific circumstances but this remedy is only effective for a decision-maker, not for an experimenter. We furthermore show that belief and performance do not match when conditioned on task difficulty – as is common practice when plotting the psychometric curve – highlighting common pitfalls in previous neuroscience work. Finally, we demonstrate that miscalibration and the hard-easy effect observed in humans' and other animals' certainty judgments could be explained by a mismatch between the experimenter's and decision maker's expected distribution of task difficulties. These results have important implications for experimental design and are of relevance for theories that aim to unravel the nature of meta-cognition.

## Introduction

In an uncertain and ambiguous world, effective decision making requires computing one's certainty about all decision-relevant evidence. For example, consider driving on the highway while running late for a job interview. Driving too fast would result in a very high cost if hit by another car. Driving too slowly, on the other hand, could result in losing the job. Thus, a good policy to follow is to accumulate evidence about the surrounding traffic to minimize the expected personal cost of an accident, evaluated based on ones certainty, while balancing the loss of time to accumulate this evidence. In general, decision certainty plays an essential role in value-based decisions, and is thus an essential component of every-day decision making. There exists a large body of evidence that humans and animals encode such information, which allows them to feature a belief, or confidence, about the correctness of their decisions (a process sometimes referred to as meta-cognition) [1]–[10]. It is important to mention that in this paper it is not claimed that belief is explicit, conscious or readily accessible for verbal report. Rather, belief can be implicitly coded (e.g., a function of several variables of the decision process), unconscious in many cases and difficult - if not impossible - to access verbally.

Nevertheless, for the decision maker, such belief is important as predicting the decision's outcome and monitoring her task performance are only effective if this belief is correctly reflected in the decision maker's performance. The relation between belief and performance is also essential for an experimenter who wants to assess the decision maker's belief to gain insight into her decision making strategy [5], [8] by, for example, using the decision maker's performance as a proxy for her belief [11]. In both cases, belief and performance are assumed to be closely related or equivalent.

Assuming that the decision maker holds the correct model of the world, it is intuitive that her belief should equal her performance [12]. For instance, if a subject is correct 80% of the time across trials of a particular experimental condition, it seems logical to conclude that, on any given trial, the subjects should believe that her chances of being correct is 80%. Indeed, some previous studies on decision making have implicitly assumed these measures to be similar [5], [8] or even exchangeable [11]. Surprisingly, however, we show that belief equals performance only for a very narrow family of tasks and decision strategies. So, if a subject has the correct model of the world, how is it possible that her belief does not correspond to her performance in most realistic conditions? And if that is the case, how can subjects trust their belief to monitor their performance in order to improve it in any given task?

The theory that we outline below reveals (i) the correct variables that a decision maker should monitor during a task, (ii) the conditions under which an experimenter (that is, and external observer controlling some variables of the task at hand) can measure belief at each trial or on average, and (iii) the correct performance measures to be used to estimate the decision maker's belief without bias and with the least possible variance. Our theory is based on a normative view of the decision-making process, in which the decision maker utilizes the correct model of the world to infer optimal decisions given all available evidence. To this respect, our approach differs from comparable, but heuristic explanations for human and animal confidence judgments [8], [13]–[15] that might employ comparable mechanisms but do not have the same ideological underpinning. As such, our theory provides an upper bound on the relation between belief and performance. Despite this, we demonstrate some significant deterioration of this relation, which can, due to deviations from the normative ideal, only worsen in practice. Based on these findings, we point out some pitfalls in previous neuroscience work, we provide a new hypothesis for the origin of the hard-easy effect, and we present a different perspective on models of confidence miscalibration [16]–[18].

We first introduce the general formalism, based upon which we define belief and performance. This is followed by discussing their relation and showing that they are rarely equivalent. We then focus on the more specific case of diffusion and race decision making models, and demonstrate how our general findings apply to these two model types. After that, we discuss the consequences of these findings to both the decision-maker and the experimenter observing this decision-maker, focusing on the relation between the psychometric curve and the decision maker's belief, and the hard-easy effect in human confidence judgments. At last, we put our findings into the more general context of previous work.

## Results

### Formalism

**Figure 1:**
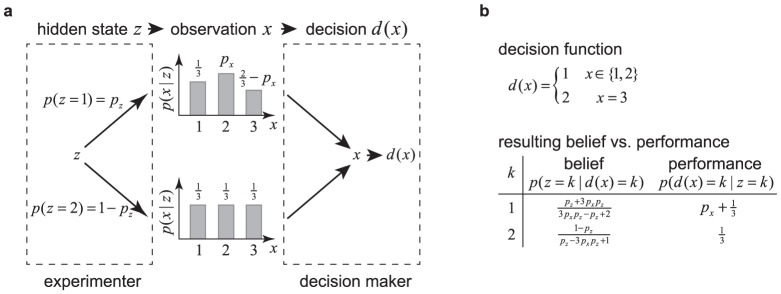
Illustration of framework with a three-sided coin example. (a) In each trial of a sequence, a hidden state 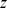 is picked by the experimenter, based on which the observation 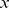 is generated. The decision maker only observes 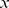 but not 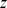 and chooses option 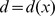 where 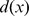 is a deterministic function that maps observations into decisions. In this 2-AFC example there are two possible hidden state, causing 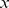 to be sampled either according to a biased 3-sided coin 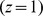, or a fair 3-sided coin 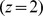. (b) For the given decision function, which maximizes the number of correct decisions for 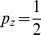 and 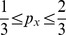, the resulting belief and performance are shown for either choice/hidden state. Belief and performance only match if 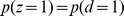, that is, when 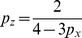.

In general, we consider 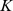-alternative forced choice (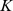-AFC) tasks (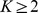) with a sequence of independent trials, in each of which an experimenter determines the hidden state 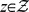 of the world, and the aim of the decision maker is to identify this state based on limited information (Fig. 1). At the beginning of each trial, the experimenter draws the hidden state 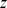 from the prior probability distribution 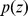. This state can take one of 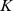 values out of the set 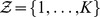. Consider, for example, an orientation categorization task, in which a displayed orientation is generated stochastically from one of two categories, and the decision maker's task is to identify this category upon observing the orientation. In this example, we would have 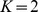, such that the generative category 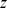 can take values out of the set 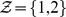. Furthermore, if each category is a-priory equally likely, we would have 
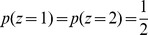
.

The decision maker does not have direct access to the hidden state 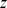, but instead observes some 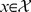 (for example, the displayed orientation) that is stochastically related to 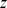 by the generative model 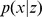 (how the experimenter generates orientations for each category). Based on the observation 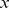, which might represent sensory input (the image of the displayed orientation on a screen) or neural activity (the firing rate of orientation-selective neurons in area V1), the decision maker commits to the choice 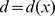 by utilizing the deterministic decision function 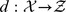 (we will write 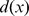 whenever we need to be explicit about its relation to 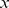). Thus, we assume that all stochasticity from the decision maker's choices has its origin in the stochasticity of how observations are generated from the hidden state (but see Generalizations). In that sense, what we called *observation* is similar to the *decision variable* in Signal Detection Theory [19], and our decision function 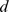 is a generalized version of the threshold that the decision variable is compared to. In addition to a deterministic decision function, we assume that the decision maker knows (for example, through experience) both the prior 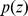 and the generative model 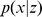, such that she could, for example, employ the decision function 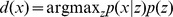 that maximizes her posterior belief 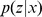. In our orientation categorization example, this would correspond to choosing always the category that was the most likely to have generated the observed orientation. While this might be a sensible function to use in general, our exposition is also valid for any other arbitrary choice of the decision function.

We will consider situations in which the experimenter has no or only limited access to the observation 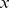 as perceived by the decision maker. For example, 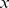 might represent the decision maker's neural activity in response to the displayed orientation, and the experimenter only observes the decision maker's choices, as determined by 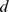. One could also imagine that the experimenter only has control over the generative category, is unable to observe the stimulus orientations in individual trials. In both cases, the experimenter cannot know 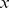 with certainty as many different values of 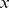
could lead to the same decision 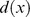. More specifically, we will differentiate between two cases: (i) the experimenter has no access to 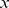 and only observed the decision maker's choices, 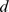, or (ii) the experimenter has partial knowledge of 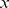 (to be defined more precisely later).

To illustrate our task setup further, consider a simple 2-AFC, in which the experimenter chooses at each trial the hidden state 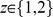 according to 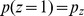 and 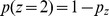 (Fig. 1a). Based on this, one of two 3-sided coins (one fair, one biased) is chosen to generate the possible set of observations 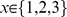, either from coin 1 by 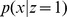, or from coin 2 by 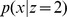
(see Fig. 1a for generative probabilities, parameterized by 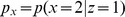; 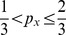). The decision maker observes the outcome of this coin flip, but does not know which coin was used to generate it. Assuming 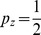 and 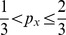, it is easy to show that the optimal strategy is to pick coin 1 (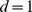) if 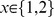, and coin 2 (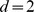) otherwise (Fig. 1b, this corresponds to the maximum a-posterior estimate of the coin state; with 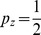, 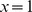 does not reveal anything about the hidden state, such that 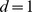 was chosen arbitrarily in this case). The experimenter, in contrast, only observes this decision, 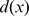, but not the outcome 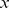 of the coin flip. This abstract task contains all the essential ingredients of our framework and will be used throughout the text to illustrate important concepts.

### Relating Belief and Performance

To relate the belief of the decision maker to the performance observed by the experimenter, let us first define what exactly we mean by these measures. The ‘belief’ refers to the decision maker's belief at decision time of choosing the correct option [20]. Thus, given observation 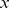 and potential choice 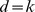, this belief is the probability 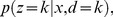
(1)


Here, we explicitly condition on the decision 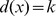 to make clear that we only consider observations 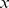 that lead to decision 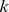. This conditioning is only hypothetical (“what is my belief if I were to choose 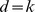
”), such that the belief can be computed before a choice is performed. For the same reason, our analysis is easily generalized to the belief of un-chosen options, but to simplify exposition we restrict ourselves to the option that is finally chosen. In either case, the belief is a subjective probability, and available to the decision maker in every single trial.

The experimenter measures the decision maker's performance by the fraction of times that the correct choice was made. Thus, for a given hidden state 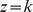, and assuming no knowledge of 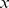, the experimenter measures the probability that the decision maker chose 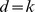, that is 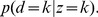
(2)This performance measure is standard in the psychophysics and perceptual decision making literature [5], [8], [21]. It is a frequentist probability estimated by averaging over many trials in which 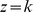, that is, trials in which the stimulus is maintained constant. This is, for instance, the measure that is plotted in psychometric curves for 2-alternative forced choice (2-AFC) task.

Given these definitions, we want to address how performance measured by the experimenter (Eq. (2)) relates to the decision maker's belief (Eq. (1)). As an intermediate step, we will first explore the condition under which performance equals belief 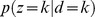 averaged over observations 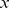, given by 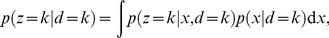
(3)where the integral is over the full support of 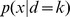, that is, all possible values of 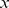 that lead to choice 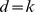. A joint probability decomposition of 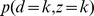 reveals that 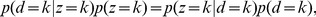
(4)where 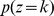 and 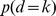 are the fractions of trials that the hidden state was 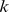, and 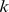 was chosen, respectively. This equality shows that the performance is only equal to the average belief, that is 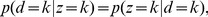
(5)if 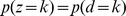. In other words, Eq. (5) is only true when the frequency of choosing 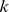 equals that of it being the correct choice. This is not always the case. For instance, these two probabilities differ in our 3-sided coin example (Fig. 1a), when choice 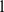 is correct with probability 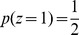 and 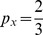. In this case, if subjects pick the most likely choice, they will pick choice 1 with probability, 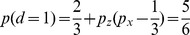. Clearly, 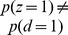, because choice 1 only occurs on 50% of the trials (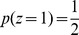), but is picked by the subject over 83% (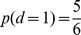) of the time. As a result, the decision maker's average belief will differ from the performance measures by the experimenter. In general, 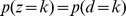 might hold for symmetric tasks with uniform priors over hidden states, but is likely to be violated in tasks that are asymmetric (for example, Fig. 1), or in which some choices are more likely to be correct on average than others.

To summarize, belief only equals performance when the frequency of choices matches the frequency of them being correct, and even then, this belief is the average belief across trials (Eq. (3)) in which a particular choice was made.

### Accumulation of evidence over time by diffusion/race models

Even though the established formalism is already able to capture simple experimental setups, its applicability is limited to cases where all the experimenter observes are the decision maker's choices, and nothing else (that is, the experimenter does not have access to 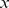). In general, the experimenter might have access to further information, such as the reaction time, that reveals additional details about the decision maker's state at decision time. Consider, for instance, a situation where the observation 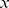 is a noisy version of an image drawn by the experimenter. In this case, clearly, the experimenter will have some, but only partial information about the decision maker's observation. A second important limitation of previous examples is that we have assumed the observation 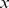 to be immediately available, whereas, usually, the decision maker needs to accumulate evidence over time before committing to a decision. In this and the next section we extend the previous formalism to fully accommodate in the theory these situations. In the following, we focus on diffusion and race models due to their popularity in cognitive sciences and neuroscience and their mathematical tractability. Despite this, we want to emphasize that our general theory on the relation of belief and performance remains valid even if the particular assumptions underlying these model choices (such as independent and identically distributed momentary evidence) are violated.

**Figure 2:**
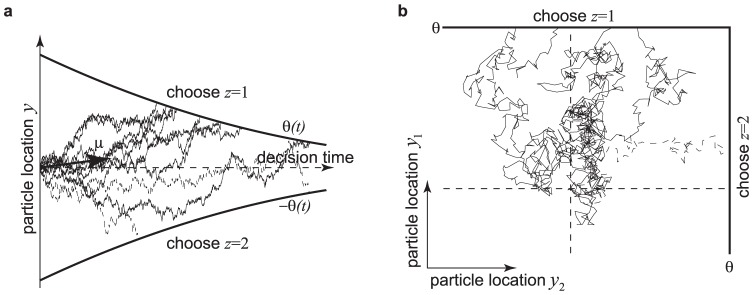
The diffusion model (DM) and 2-race model. (a) In a DM, a particle drifts and diffuses over time. A decision is performed as soon as this particle reaches one of the two boundaries. The mean drift rate 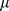, which is unknown to the decision maker, determines which of the two choices is correct. In this illustration, the drift is towards the upper boundary, corresponding to hidden state 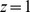, such that 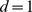 is the correct choice. We show eight (solid) trajectories leading to the correct choice (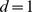) and two (dashed) trajectories leading to the wrong choice (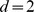). Our framework allows for time-varying boundaries, as shown here and used to generate Figs. 3a/b and 4a/b. (b) A race model features 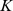 races (here 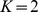) that compete against each other in a race towards a boundary of height 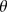. The race that first reaches its associated boundary determines the decision. The set of all races is described by a drifting/diffusing particle in 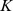-dimensional space. In our illustration this particle drifts towards the upper boundary (thus 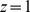) and diffuses in both dimensions. Thus, four (solid) trajectories lead to the correct choice (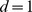), and one (dashed) trajectory leads to the incorrect choice (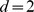).

We start by considering a 2-AFC random dot reaction time task [22]–[23]. At each trial, the experimenter chooses the motion direction (left or right) and coherence (fraction of dots moving coherently) which is subsequently used to generate the visual stimulus. The decision maker is told to identify as quickly and as accurately as possible the motion direction. In this task, the hidden state 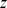 is the motion direction, while the coherence is a nuisance parameter that does not carry any information about the correct choice. The momentary evidence about 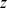 in a short time window 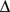 follows a Gaussian 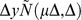 with mean 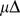 and variance 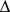. Its mean rate 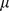 is determined by the experimenter, and is positive for left-ward motion (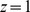) and negative for right-ward motion (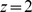), and its magnitude 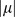 is proportional to the coherence of the random-dot motion. The decision maker can infer 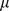 through the momentary evidence 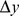, which she can accumulate over time by a bounded drifting and diffusing particle 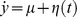 with 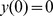, where 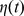 is a unit variance Gaussian white noise [24]–[27]. In this diffusion model (DM, Fig. 2a), 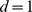 is chosen if this particle hits the upper, potentially time-varying boundary at 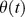, that is 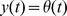, and 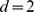 is chosen if it hits the lower boundary at 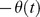. We allow these boundaries to change with time to demonstrate the generality of our framework. Clearly, all principles discussed here transfer immediately to the more standard case of time-invariant boundaries. At the point when either of the boundaries has been reached, all the information required to compute the belief about the hidden state 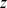 is the particle location at this time, that is 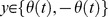, and the decision time 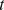 (see Methods: 2-AFC decision making with diffusion models) [5], [26]. Thus, we define the observation 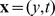 as the pair particle location at decision and decision time, which are the sufficient statistics of this belief. In such a setup, the experimenter might be able to observe the time 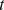 of this decision, but not necessarily the true state of the variable 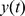. This gives the experimenter partial knowledge of the state of the DM because knowing decision time 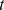 tells the experimenter that one of the two bounds has been hit. More formally, knowing the decision time 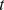, the experimenter can restrict 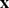 to the set 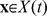, which denotes the set of observation vectors 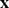 with decision time equal to 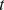 which is simply the set in which the first component of the vector 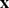 is either 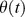 or 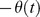. In fact, the experimenter can also infer whether the positive or the negative boundary was hit from observing the response of the subject, although the value of the boundary itself remains unknown. This partial knowledge can be exploited by the experimenter to get a better handle on the decision maker's belief, as we will describe further below.

The same logic applies to scenarios in which more than two options are available to choose from. Let us consider a 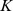-AFC task for 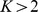 (Fig. 2b for 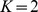). In this case, we assume that the experimenter presents a stimulus that determines 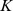 non-negative drift rates 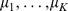. The hidden state is determined by the largest of these rates, such that 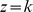 if and only if all races 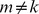 feature a lower drift rate than race 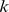, that is, 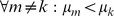. The decision maker observes 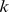 races, given by the drifting/diffusing particle 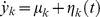 starting at 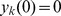, towards a potentially time-varying boundary 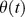 starting at 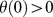. A decision strategy that maximizes the posterior belief under certain circumstances is to choose 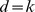 if race 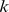 is the first to reach this boundary (see Methods: *K*-AFC decision making with race models). That is, 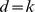 if and only if 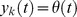, where 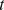 is the first time at which either race has reach the boundary. Independent of the used decision strategy, it can be shown that the sufficient statistics that completely determine the decision maker's posterior belief about the hidden state are time 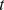 and the particle locations 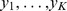 at this time (see Methods: *K*-AFC decision making with race models) [27]. Thus, we define an observation in the race model setup to be these statistics at decision time 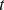, that is 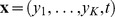, where decision 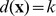 corresponds to 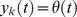 and 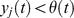 for all 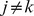. The experimenter can again observes both the chosen option and the time of this choice, and so has partial access to the decision maker's observation 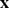 by 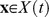, where 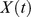 denotes all possible race states that result in a decision at time 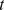 (which are all the vectors 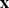 in which one of the first 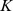 components is equal to 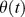). These examples illustrate that, despite our conceptually simple task formulation, we are able to capture a wide range of possible tasks and decision mechanisms that include non-uniform priors, and decisions that require the accumulation of evidence whose reliability might vary across trials.

### Relating belief and performance for partial knowledge of the observation

In the preceding cases, the experimenter has partial knowledge of the observation through observing the decision time. Here we describe how this information is used to refine the previously established relation between belief and performance. In general, we assume that partial knowledge of 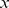 can be expressed by 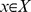, which indicates that the experimenter knows that the observation has some features shared by all observations in 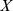 (like, as the previous cases, the decision time), but does not know the observation 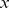 itself. As a consequence, the performance as measured by the experimenter is given by 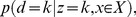
(6)where, when compared to Eq. (2), we additionally condition on 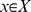. Hence, we assume that the experimenter evaluates the performance by binning trials by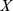. Setting 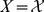 (where 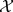 is the set of all values that 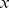 can take) recovers the original case in which the experimenter was unable to observe 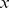, demonstrating that the partial information case strictly generalizes the original case.

To relate belief and performance if partial knowledge is available, we again decompose the joint probability 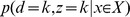 to get 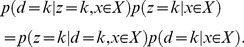
(7)Thus, as before, performance only equals the average belief if 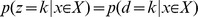, that is, if the fraction of choosing 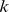 in trials in which 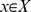 equals the fraction of this choice being correct in such trials. Furthermore, the belief on the right-hand side of Eq. (7) is 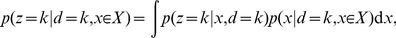
(8)which is the trial-by-trial belief averaged over trials in which 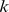 was chosen and 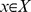 holds. The integral is over the full support of 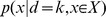, which is the subset of 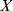 that leads to choice 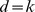. Thus the same restrictions apply to the relation between belief and performance as when the experimenter does not know 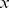, only that now they relate to the subgroup of trials in which 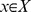.

### Belief and Performance for Diffusion and Race Models

**Figure 3:**
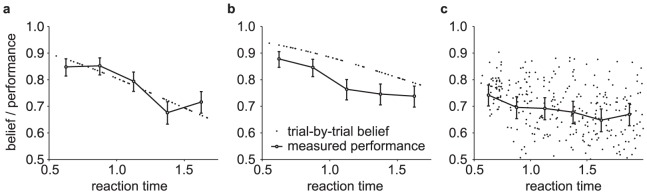
Relationship between belief and performance in diffusion models (DMs) and race models. (a) In a DM with uniform priors, 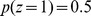, and symmetric boundaries, belief (data points) and performance (line) are equivalent. In the DM used to generate this figure, the boundaries collapse over time, causing a drop in belief/performance with time. If the boundaries were time-invariant instead, both belief and performance would be independent of time. (b) In the same DM with the same symmetric boundaries, but a non-uniform prior of 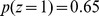, this equivalence fails to hold. It appears as if the decision maker were overconfident in her choices. (c) Simulations for a 2-race model with uniform priors, in which the winning race determines the choice, feature a strong fluctuation of the trial-by-trial belief around the decision maker's performance. It appears as if the decision maker features a belief that is idiosyncratic, fluctuating very strongly at each trial, although on average it equals her performance. In all panels the performance (with 95% CI) is estimated in bins of 250 ms, each containing data from 500 trials. The performance is measured as a fraction of trials in which option 1 was chosen when this choice was correct. For each of these bins, 10 examples (50 for the 2-race model) for the trial-by-trial belief when choosing option 1 are shown. This trial-by-trial belief is assumed to be either reported by the decision maker, or to be estimated from neural population activity. Details of how the models were simulated are in Methods: Generating Figures 3 and 4.

Returning to the example of the diffusion model, the decision maker's belief when choosing option 1 at time 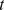 is 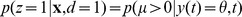 (where observation 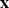 is defined as 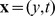) the performance measured by the experimenter is 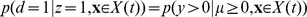. Here 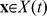 denotes that the experimenter knows that a decision has been made at time 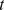, and 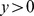 implies – without specifying the height of the boundary – that option 1 has been chosen. We furthermore assume a symmetric prior on the drift rates, that is, 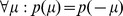. This implies for any decision time 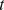 a uniform prior on hidden states, 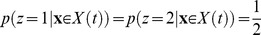, and an equal probability of choosing either option, 
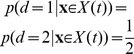
, such that the probability of choosing either option equals to it being correct, that is 
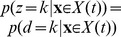
. Under these conditions we have previously established [26] that performance equals average belief, such that 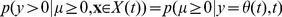
(9)Thus, the decision maker's belief when choosing option 1 at time 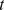 equals her probability of making a correct choice at this time (Fig. 3a). It has not been shown before, however, that as soon as we start introducing asymmetry into the task by, for example, a non-uniform prior, this relationship will break down (Fig. 3b).

Interestingly, the belief averaged over all decisions made at time 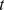 (Eq. (8)) in this example turns out to be equivalent to the belief held by the decision maker in each of these trials (Eq. (1)). Indeed, using our more general notation to express this, we have 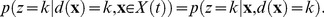
(10)Thus, if the experimenter bins trials by decision time and computes the percentage of correct choices in each of these bins (as in Fig. 3a), this percentage will correlate perfectly with the decision maker's trial-by-trial belief at these decision times. In this model, the perfect correlation arises from to the lack of variability in decision confidence in this model, a result that will be violated in most general models (see below).

To understand why this property holds, it is instructive to revisit Eq. (8), which states that the average belief is the trial-by-trial belief held by the decision maker averaged over all trials in which choice 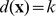 was made, and 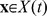 specifies the time of this choice. For the diffusion model, knowing both choice and decision time corresponds to knowing which of the two boundaries was reached, and at which time, thus specifying the observation by 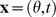 and 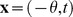 for 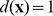 and 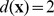, respectively. Therefore, even if the bound height 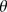 and thus the exact value of 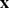 is unknown, the experimenter's knowledge of decision time and choice restricts 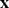 to a single possible value, which results in the same belief every time this choice is made at this time. In general, as long as 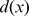 and 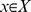 restrict 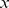 to a single possible observation, Eq. (10) holds. As a result, the diffusion model has the fortunate property that the experimenter has access to the trial-by-trial belief solely by measuring the performance of the decision maker. This has an important implication: for DMs applied to symmetric 2-AFC tasks, trial-by-trial belief, and not just averaged belief, equals performance, which is a very useful property for experimenters interested in inferring belief from performance [26].

This property is not shared by multiple-race models (Fig. 3c). In a multiple-race model as described above, the belief of the decision maker when choosing option 1 at time 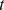 is her belief that the drift of the first race is larger than that of all other races, as given by 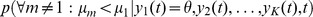, where we implicitly condition on no race having reached the boundary before 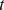. The performance as measured by the experimenter is the probability that option 1 was chosen at time 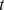, given that it was correct, as specified by 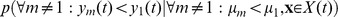, where 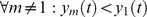 implies that race 1 is the first to reach the boundary without specifying this boundary's height, and 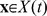, where 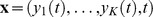, denotes that some decision has been made at time 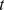. We furthermore assume that the prior 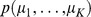 has the same density for all permutations of the indices 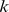 on the 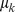
's, such that 
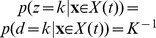
 for all 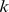. Under these conditions, we can again relate performance and average belief by 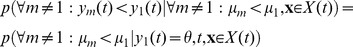
(11)However, in contrast to the DM, the average belief, on the right-hand side of Eq. (11) is not equal to the trial-by-trial belief as held by the decision maker. This discrepancy stems from the decision maker's belief not only depending on the state of the winning race, but also on that of all other races. For example, all races being close the boundary would induce higher uncertainty about the correctness of the decision than if there is a clear separation between the winning and the losing races (see also Eq. (25)). As a result, this belief varies across trials even if the same decision is made at the same time. Thus, the experimenter is unable to determine the decision maker's trial-by-trial belief by measuring her performance, but only its average. More formally, the probability 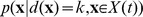 that specifies in Eq. (8) which trials the belief is averaged over, now has non-zero probability for multiple values of 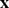. This is because 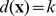 and 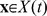 specify the winning race and bound-hitting time respectively, but the state of the losing races are only restricted to be somewhere below the decision threshold. Thus, these can take any state as long as 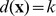 and 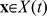 hold. As a result, the average is computed over all possible states of the losing races that satisfy 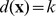 and 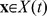, causing the average belief to differ from the decision maker's trial-by-trial belief. As we will show later, this is a general property of all decision making procedures in which the decision maker's belief depends on decision variables that are not accessible to the experimenter.

In the example in Fig. 3c, the Pearson correlation coefficient between the binned percentage of corrected trials and the decision maker's trial-by-trial belief drops from close to one for the diffusion model to around 0.18 for the 2-race model. With less than 200 trials worth of observations, such a correlation coefficient is not even considered significantly different from zero at the 0.01 level. This illustrates that, in practice, such fluctuations can seriously impair the relation between trial-by-trial belief and actual performance.

### Relevance for Decision Maker

We have established that the decision maker's performance equals her belief only in rare cases, even if we assume that the decision maker holds the correct model of the environment. For instance, if the probability of the choices is not uniform, or subjects shows biases or preferences for a particular choice, belief and performance are not expected to coincide. The equality between belief and performance depends not only on the decision maker's strategy to perform the decision (that is, the used decision model, e.g. with biases or not), but also on the task that the decision maker has to solve (e.g. with or without non-uniform priors on the correct choices). The dissociation between belief and performance in most natural conditions therefore seems to violate the very assumption that the subjects have a correct model of the world since her own belief does not predict performance.

Yet, let us reconsider the quantity that the decision maker should monitor to feature efficient behavior. A belief (e.g. 0.8) is a useful quantity only to the extent that it predicts the percentage of time (e.g. 80%) the subject will be correct every time she observes *x* and decision *k* was taken, which is simply the quantity 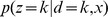. This is the same quantity we have defined as the ‘belief’ of the subject in equation (1). To compute this quantity, the subject needs to use Bayes rule, which relies on knowledge of the true generative model 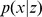 and prior 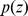. When this is the case, the belief computed by the subject will be exactly equal to 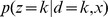, that is, equal to the percentage of time she will be correct whenever she observes 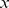 and made decision 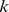. Therefore, although we have gained crucial insights into the decision process with the study of the relationship between performance and belief, the quantity we have called performance, 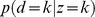, which is commonly measured by experimentalists, is not directly relevant to the decision maker's self-monitoring of her efficiency.

We can gain further insight into the sufficiency of monitoring ones belief by reconsidering the relationship we use to establish the equivalence between belief and performance. If we sum both sides of Eq. (7) over all 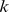, we trivially find 
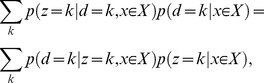
(12)showing that the average belief over all choices on the left-hand side equals the average performance over all hidden states on the right-hand side, even when 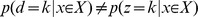, that is, even if the decision-maker does not perform frequency matching. Thus, as soon as we stop conditioning on choice or hidden state, we regain equality under all conditions. The inequality due to conditioning arose from considering a different set of trials for belief than for performance by conditioning on information unavailable to the decision maker (that is, the hidden state). Regaining equality once we consider the same set of trials confirms that monitoring ones belief will indeed provide a correct picture of ones behavioral efficiency, but only on average.

Note however that even when belief and performance are not equivalent, they are positively and linearly related *on average*. To see this, observe that in Eq. (4) both the choice probability 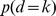 and the prior probability 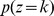 are constant across trials, such that an increase in the average belief 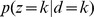 directly relates to an increase in performance 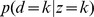. This also holds for the more general case in which we condition on a subset of observations, as in Eq. (7). As a result, the decision maker can use the average belief's gradient to improve her performance even in cases where these two quantities are not equivalent. Still, one again should be aware that this linear relationship holds only on average, such that – depending on how strongly the trial-by-trial belief fluctuates around the average belief, as shown above – this relationship might be of limited use.

### Relevance to Experimenter

**Figure 4:**
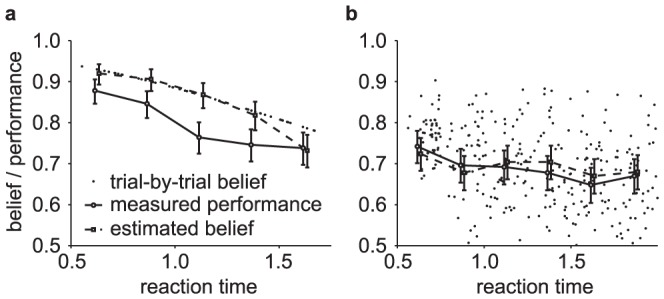
Comparing estimated belief with performance and trial-by-trial belief. (a) A DM with a non-uniform prior of 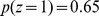 as in Fig. 3b. Trial-by-trial belief differs from performance because of the asymmetric prior. By contrast, the estimated belief using Eq. (13) matches the trial-by-trial belief, because the decision maker's state is fully observable in a DM. (b) A two race model with uniform priors as in Fig 3c. This time, the decision maker's state is not fully observable because the state of the losing race is unknown to the experimenter. As a consequence, the belief estimated by Eq. (13) no longer matches the trial-by-trial belief of the observer but only the averaged belief, where the average is performed over the state of the losing race. Details of the model simulations are described in Methods: Generating Figures 3 and 4.

From the experimenter's perspective, an equality between belief and performance is important as it would imply that one could use performance as a surrogate for belief (or average belief). Thus, experimenters might be tempted to avoid more complex experimental setups in which these two quantities are not equal, since it would become unclear how to assess the decision maker's belief. Yet, a simple remedy presents itself by considering what needs to be known to evaluate average belief directly. Average belief, 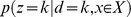, is the probability that the hidden state was 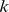 when subject chose 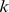 and 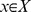. From a frequentist point of view, this is the percentage of time the subject made the correct choice (that is subject chose 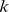 when the hidden state was indeed 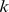) given partial knowledge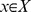. Therefore, if we bin all the trials for which the subject chose k and 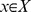, the percentage of correct responses will converge to 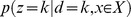 for very large number of trials. More formally: 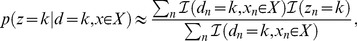
(13)where the sums are over all trials, indexed by 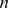, and 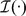 is the identifier function that returns 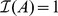 is the statement 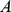 is true, and 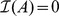 otherwise. This shows that the experimenter can evaluate the decision maker's average belief, even when belief and performance do not correspond to each other, as illustrated in Fig 4a. However, even then, this average belief might only be weakly correlated with the decision maker's trial-by-trial belief (for example, Fig. 4b), such that this average belief might tell the experimenter little about the decision maker's belief in individual trials.

To summarize, the relevant quantity for estimating belief is not performance as defined by the psychometric curves, but the percentage of correct responses conditioned on the subject response and partial knowledge of *x* (for example, percentage of correct response given that the subject chose rightward motion and the reaction time is between *t* and *t*+*dt*). In a psychometric curve, the percentage correct is conditioned on the true state of the world (for example, actual motion was to the right), while we are now conditioning on the decision maker's response. Note that this is the same fix as the one we used in the previous section when we considered the point of view of the decision maker.

### The hard-easy effect in psychometric curves

**Figure 5:**
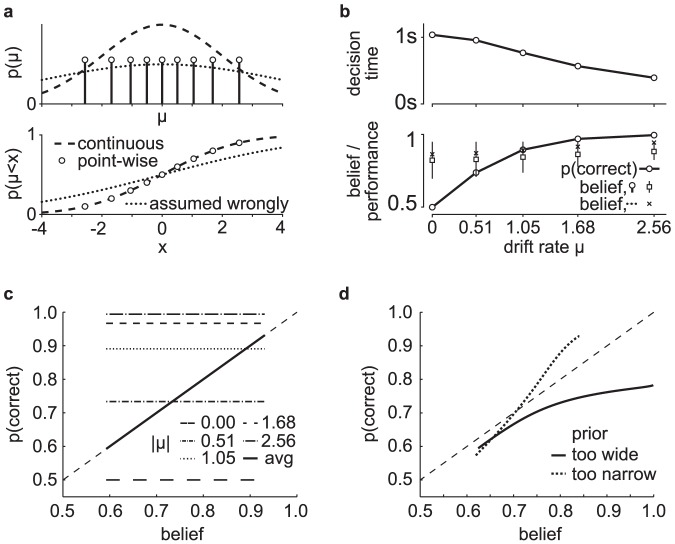
Mismatch between average belief and performance when conditioning on task difficulty: the hard-easy effect and miscalibration. We simulated a task with varying difficulty given by a diffusion model with a drift rate whose magnitude and sign varied across trials, while being constant within each trial. (a) The top graph shows the across-trials point-wise prior on the drift rate used in the simulation that roughly approximates a zero-mean Gaussian (dashed line). We computed the decision maker's belief by either using this point-wise prior directly, or by assuming it to follow a too-wide zero-mean Gaussian (dotted line). The bottom graph shows that the point-wise prior corresponds to the 10^th^, 20^th^, …, 90^th^ percentile of the Gaussian it approximates. (b) The decision maker's chronometric (top) and psychometric (bottom) function over task difficulty (magnitude of 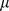) for non-negative drift rates. Correct choices here correspond to hitting the upper bound of the diffusion model if the drift rate is positive, and the lower bound otherwise. The bottom graph also shows the decision maker's average belief over 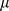 for both correct and error trials (dots exactly one top of each other, as confidence for correct and error trials is identical) based on the correct, point-wise prior (squares, +/− 2SD) and on the incorrect Gaussian prior (crosses). In both cases, the mismatch between average belief and performance when conditioning on task difficulty is clearly visible. (c) The calibration curves, showing the probability of performing correct choices as a function of the decision maker's belief. When binning trials by difficulty (that is, drift rate magnitude), this choice probability is constant while the decision maker's belief varies across trials. This results in flat calibration curves (dashed/dotted lines), caricaturizing the frequently observed hard-easy effect. Once we stop conditioning on task difficulty, the calibration curve reveals perfect calibration (solid line). (d) Calibration curves for a mismatch between the actual distribution of task difficulties and that assumed by the decision maker to compute her belief. We consider the case in which the decision maker's distribution is too narrow (that is, has too small standard deviation; dotted line) or too wide (too large standard deviation; solid line). Both cases feature a clear miscalibration of the decision maker's belief.

In general, the relation between belief and performance breaks down as soon as performance is measured conditional on events that are fundamentally inaccessible either to the experimenter or the decision maker, that is, in the case of information asymmetry. This breakdown could explain a conspicuous result known as the *hard-easy* effect: when asked to estimate their confidence in a judgment, subjects tend to overestimate their confidence on hard trials and to underestimate their confidence on easy trials [17], [28]–[29]. To see how such an effect could arise from this breakdown, let us consider a simple reaction time task, for example the random dot motion task described before, whose difficulty varies between trials. We represent this difficulty by, at the beginning of each trial, drawing 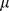 from a point-wise distribution shown in Fig. 5a, corresponding to a task in which the difficulty is interleaved across trials and can take one of a fixed number of alternatives. Here, the sign of 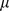 determines the hidden state 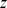, and 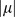 specifies the trial's difficulty (that is, the dot motion's coherence), with smaller 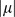
's corresponding to harder trials [26]. The range of possible 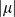
's controls the average difficulty of the task. A standard practice in such setups is to bin trials by their difficulty 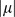 and plot the average reaction time and fraction of correct choices for each of these bins separately (the so-called chronometric and psychometric curves, respectively). Using standard analytical results for the first-passage time and choice probability for diffusion models in which 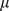 determines the drift rate (see Methods: Computing belief in a drift diffusion model with varying difficulty) leads to the chronometric and psychometric curve shown in Fig. 5b. Here, we have chosen a diffusion model with time-invariant boundaries, as the assumption of a trial-by-trial change in task difficulty causes the belief at the boundary to be time-dependent even when the boundary is not. Our conclusions do not depend on this choice, as the same principles apply to the case of time-dependent boundaries.

Intuitively, one would expect the fraction of correct choices, as shown by psychometric curve, to be a good predictor of the decision maker's belief. However, comparing it to the across-trial average of the optimally computed belief (Eq. (28), shown in Fig. 5b) reveals this to be a fallacy. More specifically, the performance varies widely as a function of difficulty, while the average belief is only very weakly related to this difficulty. This is confirmed by a correlation coefficient below 0.35 between the psychometric curve and the trial-by-trial belief.

As before, the origin of the difference between belief and performance lies in conditioning the performance measure on an event that is fundamentally inaccessible to the decision maker, in this case the trial-by-trial difficulty 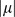 (although this time we are assuming that the experimenter knows more than the subject, as opposed to the converse). In this experiment, the decision maker does not know this difficulty, which is varied from trial to trial, and so needs to rely on the prior distribution (Fig. 5a) across trials to infer her belief. This leads to overconfidence in hard trials, and underconfidence in easy trials (left-most and right-most point in Fig. 5b, respectively). Consider, for example, trials in which 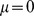 (corresponding to 0% coherence in the random dot task), such that performance is, by definition, at chance. Nonetheless, random fluctuations in the stimulus cause the decision maker to decide for one of the two options, at which point her belief about the decision's correctness will be above chance. In fact, it can be shown that a belief of 0.5 will only ever occur for the impossible case of infinite decision times (Eq (28)). As a consequence, the decision maker's belief for trials in which 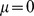 will be above her average performance in these trials, which, from the experimenter's point-of-view, leads to overconfidence. A similar argument explains the underconfidence for trial difficulties in which the decision maker features close-to-perfect performance. Thus, even though by Eq. (12) the belief equals performance when averaged across all difficulties, assessing this equality while conditioning on trial difficulty makes this equality seem violated. This last point is particularly important in the light of claims that this *hard-easy effect* might be grossly over-estimated due to simply being an artifact of binning or measuring performance by averaging over binary choices [16], [30]. In our case, it instead stems from conditioning the decision-makers reported belief and observed performance on variables that are not readily available to the subject. Although we have shown this result for a particular example of a diffusion model with time-independent decision bounds, our results are generally valid also for diffusion models with time-dependent bounds and race models. As we shown next, this effect could also arise even when performance is not conditioned on task difficulty, but the subjects assume the wrong prior over task difficulty.

### Miscalibration due to the mismatch between experimenter's and decision-maker's prior: signatures of suboptimal priors

Calibration of confidence judgments is usually assessed by the calibration curve [18], [31]–[33], which results from binning trials by the reported confidence and then plotting the fraction of correct trials for each bin. For perfectly calibrated decision makers, the fraction of correct trials ought to correspond to their confidence, in which case the calibration curve follows the identity line. If we perform the same analysis on the simulated behavior conditional on task difficulty in the example described in the previous section, we find strong deviations from this identity line that reflect the corresponding over- and underconfidence for easy and hard trials, respectively (Fig 5c, dashed/dotted lines; compare belief with performance in Fig 5b, bottom). In contrast, if we cease to condition on difficulty and analyze the whole dataset at once, we find perfect calibration (Fig. 5c, solid line), as predicted by Eq. (12). This again demonstrates that, as long as the belief is computed from the correct generative model (that is, in a Bayes-optimal way), average belief will equal average performance.

If a Bayes-optimal model of decision making produces perfect calibration, it follows that a calibration mismatch implies that subjects deviates from Bayes optimality. There are several methods available for detecting such deviations. For instance, in the *decision variable partition model* [32]–[33]. the experimental data are used to estimate the function employed by the decision maker to map internal observations, ***x***, onto belief. This function can then be compared to the Bayes optimal function to determine whether subjects are miscalibrated (see Methods: Modeling miscalibrations by the decision variable partition model). The problem with this approach is that it does not provide an explanation for why subject use a suboptimal function, a problem shared by other models [7], [34].

One possibility is that subjects do not know the generative model perfectly. For example, subjects would be miscalibrated if they use the wrong prior over task difficulty. This is a very likely situation as subjects have to learn the distribution of trial difficulties used by the experimenter, a process that would take much longer than the duration of the experiments. This effect is illustrated in Fig. 5d which compares the calibration curves for a model using the true prior over task difficult and one assuming a much wider (or much narrower) distribution than the true one. In this case, the model exhibits clear deviation from perfect calibration. Therefore, miscalibration could be due in part to imperfect knowledge of the generative model. This potential explanation for miscalibration has already been suggested conceptually in [12], but here we made its statement more quantitative.

### Average versus trial-by-trial belief

One important caveat to the experimenter's access to the decision maker's belief, as for example by utilizing Eq. (13), is that this belief can only be measured on average rather than trial-by-trial. This is a result of the experimenter's inability to observe 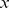 in general, causing an asymmetry in the information held by decision maker and experimenter. As shown before, the use of DMs in 2-AFC tasks do not cause such an asymmetry, as at decision time it is known that the diffusing particle has reached the boundary. In race models, in contrast, the state of the losing races is unknown, such that the belief computed with Eq. (13) does not correspond to the trial-by-trial belief (Fig. 4b) but only to the belief averaged over the unobserved state of the loosing races. As already pointed out above, this causes the trial-by-trial belief to be only weakly correlated with average performance – a correlation that might even be missed if the number of observed trials is low.

The same issues come up when considering the Sequential Probability Ratio Test (SPRT) [35]–[36] and its multi-hypothesis (that is, 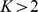) variants (MSPRTs) [37]–[40]. The SPRT, which has been shown to yield the optimal speed/accuracy trade-off for 2-AFC tasks with a single known task difficulty [36], is based on accumulating the relative evidence for one option over the other up to a time-invariant boundary, at which a decision is made. This boundary specifies the belief at decision time, such that the same belief is held every time a decision is made. In other words, the average belief at the boundary is equivalent to the trial-by-trial belief, similar to the DM. The MSPRTs, on the other hand, only feature an optimal speed/accuracy trade-off in some asymptotic sense. They exist in several variants that are all based on continuously updating the posterior belief of all options but differ in how they specify the decision bounds. Variants that commit to a decision as soon as the highest posterior belief across options has reached a pre-set threshold [37]–[38], [40] will feature the same belief across all trials, just as the DM. In contrast, if their decision threshold becomes a function of the beliefs for various options [39]–[40], their belief in the correctness of the chosen option might vary across trials, as in race models.

In general, the trial-by-trial belief differs from the average belief as soon as the minimal sufficient statistics of the decision maker's belief fluctuate at decision time, even if the experimenter bins trials according to all available information, such as choice and decision time (for a more formal statement see Methods: Equivalence of average and trial-by-trial belief). For DMs, the sufficient statistics are fully determined by the aforementioned measures, but for race models these measures are not sufficiently restrictive. It might seem that this is due to the larger number of possible choices for the race model. However, it is erroneous to attribute the difference between DMs and race models solely to the number of choices. Consider, for example, an orientation categorization task in which the observed orientation is a noisy instantiation of the orientation associated with one of the 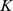 generative categories. In this case, the minimal sufficient statistics is the perceived orientation, which can be represented by a scalar value. Even if we increase the number of possible categories and with it the number of possible options to choose from, the dimensionality of the minimal sufficient statistics remains unchanged (see Methods: Minimal sufficient statistics in an orientation categorization task). Rather, what matters is the number of independent sources that ambiguously generate the observations. While in diffusion models, only a single such source exists, a race model with 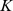 races assumes 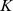 such sources. In the categorization task, in contrast, the sole source of information is the observed orientation, which does not depend on the number of possible choices. Thus, if the experimenter aims at estimating the decision maker's trial-by-trial belief, it is important to design experiments that control and restrict number and nature of these sources.

### Generalizations

Our findings are robust to changes in the details of the framework. One could, for example, imagine that the decision is stochastically rather than deterministically based on 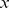 through 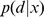. Furthermore, we could assume that the experimenter has partial knowledge of 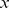 through a two-step generative model, 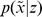 (for example, a generated image) and 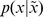 (for example, the neural response to that image), where the experimenter observes 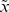 and the decision maker makes a decision based on 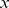. While either of these modifications changes the details of the formulation, belief still only corresponds to performance if task and prior are symmetric, and is in most cases only measurable by the experimenter on average.

Extending the framework to value-based decision making might be possible, and is mandatory for a complete theory of belief and its relation to choice. However, assigning different values to different choices introduces ambiguity about which decisions ought to be considered correct and which are incorrect. Thus, several definitions of belief and performance might be possible. For this reason, we restricted our exposition to the case in which a clear definition of “correct” and “incorrect” exists.

## Discussion

We have described how the performance of a decision maker (defined as the fraction of correct responses given the world's true state) relates to its belief of having made the correct decision, and the relevance of this relation for both the decision maker's self-monitoring and an experimenter interested in the decision maker's belief. Specifically, we have shown that performance only equals belief in cases where these measures are conditioned on quantities that are known to both the experimenter and the decision maker. This equality starts breaking down in case of information asymmetry between decision maker and experimenter. One such asymmetry occurs if the experimenter conditions performance on the true state of the world, which is unknown to the decision maker. In this case performance only equals belief for symmetric tasks, in which the probability of choosing a particular option equals the probability of this choice to be correct. Even then, the equality only holds for the average belief across many trials, while the decision maker's belief per trial might fluctuate around this average. This is the result of another information asymmetry, in which the experimenter is unable to access the decision maker's internal state at decision time, and so has to average over it. Furthermore, we have discussed that the decision maker can evaluate how well she performs the task even if her belief does not equal her performance. This is because the relevant quantity for self-monitoring is belief, computed as the expectation that the decision maker was correct given her response, rather than performance, computed as the fraction of times a decision maker was correct given the state of the world predetermined by the experimenter. Also, the experimenter does not need to measure performance to assess the decision maker's belief, as the latter is directly measurable at least on average as the fraction of times that the decision maker was correct given her choice, assuming that the decision maker has the correct model of the task. Similarly to the relation between belief and performance, however, this belief can in most cases only be computed on average, around which the decision maker's trial-by-trial belief fluctuates.

To relate belief and performance, we have assumed the decision maker to have fully learned the generative model of the task. In other words, the decision maker is able to infer optimally the posterior distribution over each of the choices being correct. While this might be a valid assumption in well-trained, low-level tasks, such as detecting a flash of light in an otherwise dark room, it is most certainly violated in more complex, high-level, decision making [41]–[42]. As we have seen, partial learning of the generative model of the task could lead to a mismatch between belief and performance, and could explain in particular the hard-easy effect (i.e. overconfidence for near-chance performance, underconfidence for high performance). This effect might arise in particular from assuming that the prior distribution over task difficulty is wider than it really is.

We have also seen that, even for rational decision-makers with a perfect knowledge of the task, the hard-easy effect arises naturally if the experimenter conditions performance and belief on trial difficulty when plotting the psychometric curve: as shown in Fig. 5b, rational decision-makers will seem underconfident in easy trials, and overconfident in difficult trials. We have identified this mismatch to result from the experimenter conditioning on variables of the task (as trial difficulty 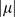 in diffusion models) that are fundamentally inaccessible to the decision maker, who instead can only rely on her prior over trial difficulties. Thus, the mismatch emerges again due to an information asymmetry between decision maker and experimenter.

Therefore, the hard-easy effect could be due to either subjects using the wrong generative model, or the experimenter assuming more knowledge than is available to the subject. Our proposal differs from a related one in [18] where the hard-easy effect is explained by subjects assuming a single, certain, but biased task difficulty. We, in contrast, assume that the subject's uncertainty about this task difficulty is to blame.

For all of the above we want to emphasize that most of the literature on the calibration of confidence judgments is based on explicit, e.g. verbal, reporting of this confidence [17]–[18], [31], [43] which could also contribute to miscalibration of confidence. There is indeed clear evidence for the existence and use of uncertainty information about task-relevant variables in multisensory information [3], [44], post-decision wagering [5], and related paradigms [20]. However, it is less clear if this information is accurately accessible for explicit reporting, or if this reporting is not part of the normal decision-making repertoire, but instead needs to be learned as a separate task, thus justifying models with a confidence judgment process that is at least to some degree separate from that leading to decisions [15], [45]–[46]. Either option might introduce additional biases [16], [30], such that it remains to be seen if the observed deviations from perfect is a property of the underlying inference process leading to the decision maker's belief, as we have suggested, or simply a property of the mapping of confidence onto explicit reports. In light of this, it seems advisable to assess this belief more directly by behavioral measures rather than by explicit reports.

Having identified some of the possible fallacies that can occur when relating belief and performance, we can revisit previously mentioned illustrative work on the decision maker's belief. In [5], for example, it at first appears as if the authors wrongly condition on the task difficulty (in their case, coherence of the motion stimulus) when relating belief and performance (for example, their Fig. 4). However, as they compute the model's belief explicitly under the assumption of an unknown task difficulty, their performance predictions for different difficulties and its relation to the observed performance for these difficulties are in fact correct. The work in [8], in contrast, attempts to establish a direct relationship between the psychometric curve, conditional on task difficulty (the odor mixture ratio in their Fig 1c/d), and the decision maker's belief, as encoded by neurons in the orbifrontal cortex (their Fig. 2). As we have seen previously, this is the kind of situation in which belief and performance are not equal because performance is conditioned on task difficulty while task difficulty is unknown to the subject. This mismatch necessarily leads to miscalibration as illustrated in Fig 5b. Fortunately, the qualitative results of this particular study did not rely on a perfect match between belief and performance, but merely on a significant correlation between these two measures, which is likely to be true in their task, even if this correlation might be weak. A similar problem occurs in [11], where the decision maker's confidence is directly derived from the psychometric curve (their Fig. 2a), again conditional on task difficulty (the width of the line that needs to be compared to a memorized reference), and is subsequently used as a parametric regressor in the analysis of functional magnet resonance imaging data. As we have demonstrated, there is no guarantee of a strong correlation between the psychometric curve and the decision maker's confidence, as for example demonstrated by a correlation coefficient below 0.35 between trial-by-trial belief and performance in Fig. 5b. Therefore, with this type of experiments. regressing performance against voxel activation only provides a weak test of whether an area is involved in encoding confidence. It is preferable to use instead a task in which the correlation between belief and performance is stronger, such as 2-AFC task in which subject knows the difficulty of the trial. Overall, these three examples demonstrate that the problems we have identified when relating belief and performance are not just obscure theoretical constructs, but occur in recent work in the neuroscience literature and have consequences for experimental design.

From the point-of-view of designing decision making models, our findings about the relation between belief and performance illustrate that models that aim to explain how humans and animals perform perceptual decision making should mostly focus on the encoded belief rather than on their performance. As long as they implement the correct generative model for the task, this belief will lead to the correct assessment of the model's task performance. For example, in both diffusion and race models, significant emphasis is put on expressions that describe the choice probability given some value of the hidden state, that is, the predicted performance [47]. Instead, one should focus on the belief, which is the relevant quantity for the decision maker. A further advantage of this change of focus is that belief can be expressed analytically even for complex time-changing boundaries and arbitrary priors (see Methods: 2-AFC decision making with diffusion models), where no expressions for performance are known [27]. This simplifies the experimental validation of such models, as has been previously demonstrated in [26].

A further contribution of our work is to show that the decision maker's belief can in most cases only be measured on average, across many trials in which the decision maker's trial-by-trial belief might differ. The form of the average depends on one hand on the decision strategy of the decision maker (for example, diffusion model vs. race model) and on the other hand on the task setup. Being able to only control the latter, experimenters should thus attempt to avoid tasks in which measuring the decision maker's belief is important and trial-by-trial fluctuations around the measurable average can cause this measure to be only very weakly correlated to the belief in individual trials. This is, as we have established, to be expected in tasks with high-dimensional sufficient statistics of the decision maker's belief. Alternatively, the experimenter needs to commit to collecting data for a large number of trials to achieve a robust estimate of the decision maker's average belief despite strong trial-by-trial fluctuations around this average. A promising venue of research that would alleviate the problems of estimating belief from behavioral measurements is gathering more specific information about the decision maker's state by multiunit electrophysiological recordings of neural population activity.

## Materials and Methods

### Decision-making framework

Here, we provide a brief description of the decision-making framework. For a more comprehensive discussion of its components, see Results. We assume that on each trial, the experimenter chooses a hidden state 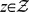 (e.g. the global direction of motion of a set of dots) according to the prior 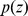. The aim of the decision maker is to identify this hidden state by means of an observation 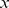 (e.g. the motion energy in the display over a short time bin, or the neural activity in area MT) that relates to 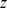 by the generative model 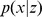, which is assumed to be known by the decision maker. In the following we show how both diffusion and race models can be described in this framework. Specifically, we derive the observation 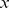 as the sufficient statistics of the posterior 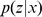, and show that the decision time allows the experimenter to gain some limited information about 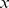 without knowing its exact value.

### 2-AFC decision making with diffusion models

In a diffusion model (DM), evidence about the hidden state 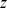 is provided in each of a sequence of small time steps of size 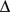 independently by the Gaussian momentary evidence 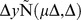 with mean 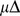 and variance 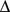. The mean rate 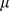 is non-negative, 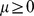, for 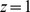 and negative, 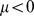, for 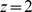. Its magnitude 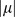 is a nuisance parameter that is uninformative about the hidden state, but determines the difficulty of the task.

We define the observation space 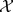 by the sufficient statistics of the posterior belief given some sequence 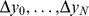 of momentary evidences from time 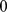 to 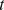 (such that 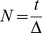) as follows. By Bayes' rule, and by the independence of the momentary evidences across time, the posterior 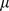 is given by 
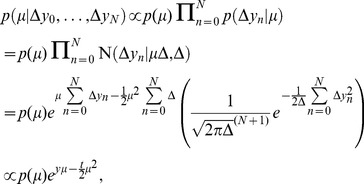
(14)where all proportionalities are with respect to 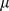, and where we have used 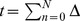 and 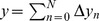. In the second-to-last line, the only dependency on the full trajectory not expressible through 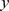 appears in the term in brackets, which is dropped in the last line, as it does not contain any 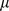-related terms. Thus, with 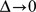, 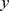 describes the location of a drifting and diffusing particle, 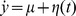. Here, 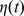 is zero-mean Gaussian white noise with 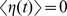 and 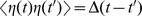, where 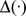 is the Dirac delta function. This shows that, independently of the exact form of the prior 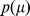, the posterior 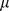 only depends on the current time 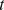, and the location 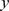 of the drifting and diffusing particle at that time, rather than on the whole particle trajectory 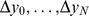. Furthermore, by our definition, we have 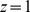 for all non-negative 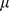, such that 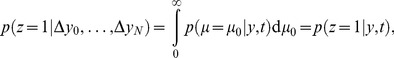
(15)which demonstrates that the decision maker's belief also depends only on 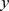 and 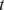, for all possible priors 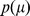. This holds even if the particle drifts in a bounded space with arbitrarily shaped boundaries [26]–[27]. Thus, if we assume decisions to be triggered at the time-varying boundaries 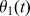 and 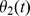 with 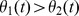 for all 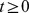, and starting at 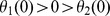, then we can define an observation by the belief's sufficient statistics at one of these boundaries. As a result, the observation is given by the pair 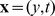, where 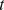 is the decision time and 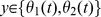. Furthermore, the set of possible observations is 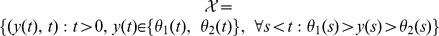, where the last condition makes sure that the particle has not crossed either boundary before 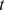. Knowing the decision time 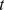 thus restricts the set of possible observations to 
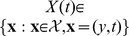
.

### 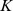-AFC decision making with race models

We assume a model with 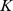 races, with race 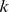 providing independent information by its associated drifting and diffusing particle 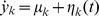 with non-negative drift rate 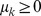, and starting at 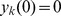. Here, the 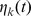
's are uncorrelated unit-variance Gaussian white noises, such that 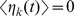 and 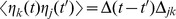, where 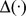 is the Dirac delta function, and 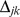 is the Kronecker delta. The hidden state is associated with the fastest race, such that 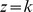 iff 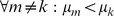. The decision maker estimates this hidden state by forming a posterior over the drift rates given the full particle trajectory of all particles. As for the DM, we find this posterior by discretizing these particle trajectories into small time steps of size 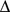, such that in the 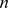 th step, particle 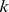 provides momentary evidence 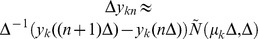. If we assume to observe these trajectories from time 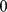 to 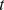, the posterior over the drift rates becomes 
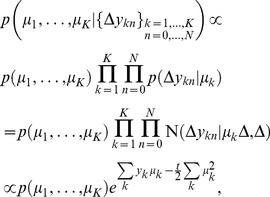
(16)with all proportionalities with respect to the drift rates, where 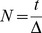, and where we have used 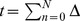 and 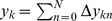. This shows that, as for the DM, this posterior depends only on time 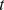 and the particle locations 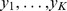 at this time, rather than the whole particle trajectory. From this posterior we find the hidden state posterior by 
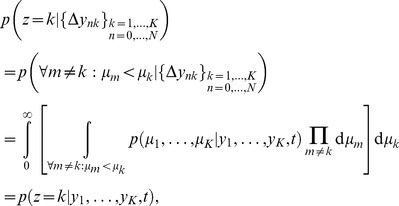
(17)which is again a function of only time and the current particle locations, thus forming the sufficient statistics of this belief. As before, the same sufficient statistics apply if the particle space is arbitrarily bounded.

A decision is made as soon as the first particle reaches a bound. If we assume that each race 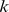 is upper-bounded independently by a time-varying boundary 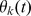 with 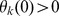, then the set of observations that describes the belief's sufficient statistics and that correspond to particle 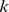 having reached the boundary first at time 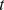 is 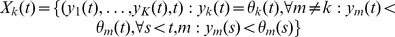, where the last condition again makes sure that no race has reached the boundary before 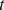. Thus, the set of observations that describe that a decision has been made at time 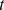 is 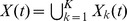, that is, the set in which exactly one of the particles has reached the boundary at time 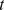. The set of all possible observations is thus given by 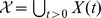. Importantly, an observation in either 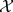, 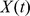, or 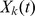 does not only describe the state of the winning race, but also those of the losing races, as the belief depends on the state of all races. In Results we assume the same boundaries 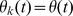 for all 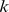 for convenience, but our formalism is also valid for boundary shapes that differ between races.

### An optimal decision strategy for the race model

Here we show that for a permutation-invariant prior 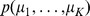 on the drift rates, and the same bound, 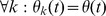, on all races, a race model that chooses the option corresponding to the winning race corresponds to choosing the option that maximizes the posterior belief. The prior needs to be permutation-invariant in the sense that it needs to be invariant to swapping the values of any two drift rates. That is 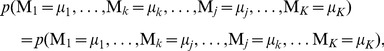
(18)needs to hold for any two 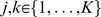, where 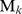 denote the random variable corresponding to the drift rate of race 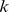. In general, this can be achieved by defining the prior as a mixture of 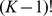 components (that is, the number possible swaps), each swapping two elements of a base distribution over 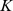 random variables. A simpler, special case of this condition is a prior with 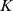 mixture components that, for each component, assumes drift 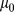 for all races except one, which instead features a drift of 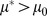. The latter prior would correspond to the case where only a single race is informative about the correct option, while all the others are equally distractive.

To show optimality of choosing the option associated with the winning race, assume that race 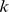 was the first to have reached the boundary 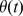 at time 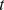. We demonstrate that, under these circumstances, the posterior belief of 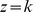 according to Eq. (17) is at least as large as for any other 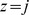 where 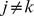. Choosing some arbitrary 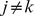, we define for the observed 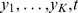, 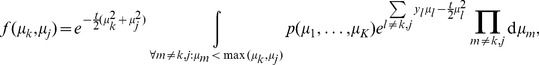
(19)which, due to the permutation-invariant prior, is a non-negative symmetric function, that is 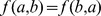 and 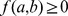. This allows us to write the beliefs of 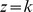 and 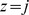 by Eqs. (16) and (17) as 
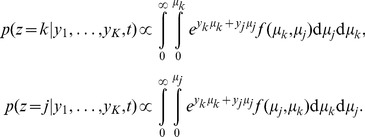
(20)Thus, in order to satisfy 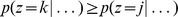, we need to have 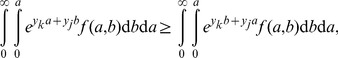
(21)where we have substituted 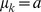 and 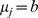 on the left-hand side, and 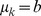 and 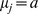 on the right-hand side. Due to the non-negativity of 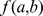 and the strictly increasing and non-negative exponential, Eq. (21) is satisfied if 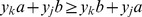 for 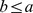 (due to the upper limit of the inner integral) and 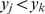 (race 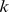 is winner, such that 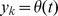 and 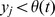). This is easily shown by using 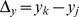, such that this inequality can be written as 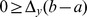, which, due to 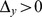 and 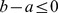, is always satisfied. As 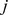 was arbitrarily chosen, it holds for all 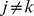, such that choosing the option corresponding to the winning race guarantees that no other choice would have led to a higher belief of being correct.

### Equivalence of average and trial-by-trial belief

After observing 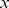 in a given trial, the decision maker commits to decision 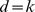, where 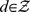, and holds belief 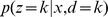. Knowing only that 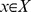, the experimenter can measure the average belief 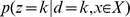 across multiple trials. As we discuss in more detail in Results, the relation between the decision maker's trial-by-trial belief and the average belief measured by the experimenter is 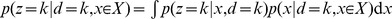, as given by Eq. (8). This relation states that the average belief is the trial-by-trial belief averaged over all trials in which option 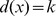 is chosen, and in which the observation 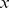 conforms to 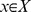. Clearly, if the set 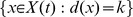 only holds a single 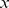, such that 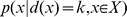 is only non-zero for this one 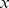, then average belief and trial-by-trial belief are equivalent. Here we consider a slightly more general condition based on the minimal sufficient statistics of the trial-by-trial belief.

Intuitively, a necessary and sufficient condition for the equivalence of average and trial-by-trial belief is that, for all trials that we average over, the observation 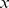 needs to lead to the same trial-by-trial belief. Thus, while it is permissible for 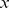 to vary across trials, its contribution to the trial-by-trial belief needs to remain constant. This contribution is formalized as the minimal sufficient statistic 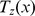 of 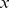 with respect to 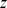, such that 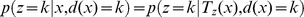. If we have two observations, 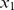 and 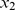, for which 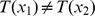, it is by the definition of minimal sufficient statistics guaranteed that 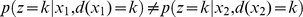. In contrast, for two observations for which 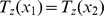 we have 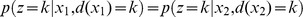 even if 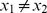. Thus, a necessary and sufficient condition for the equivalence of average and trial-by-trial belief is that for all 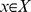 such that 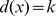 we need to guarantee that 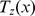 is the same constant. This condition is sufficient, because if it holds, then 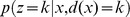 will be the same for all trials that we average over, resulting in an equivalent average belief. It is necessary, because if 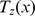 differs for at least one 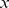, then we will average over different trial-by-trial beliefs.

To relate this condition to DMs and race models, let us consider their minimal sufficient statistics. In the case of diffusion models, these statistics are particle location and time, 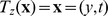. Thus, as 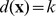 and 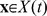 imply that a particular boundary has been reached at a known time, both 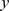 and 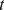 are guaranteed to be uniquely determined, such that 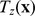 is constant under these conditions. As a result, average belief and trial-to-trial belief are equivalent. In 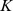-race models, on the other hand, the sufficient statistics are the state of all races and time, 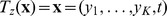. In this case, 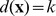 and 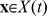 restrict time and the state of the winning race, but not that of the other races (other than them being below the boundary), such that 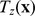 can change between trials. As a consequence, the trial-by-trial belief will fluctuate around the average belief.

### Minimal sufficient statistics in an orientation categorization task

To show that the dimensionality of the minimal sufficient statistics does not necessarily grow with the number of options available to the decision maker, consider the following task. Assume a set of 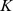 orientations, 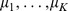, on a half-circle, 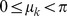, with the 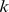
th orientation corresponding to hidden state 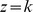. In each trial, the experimenter picks a hidden state 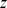 which is used to generate an oriented stimulus with orientation 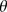 by drawing 
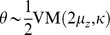
 from a von Mises distribution with mean 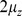 and concentration 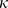. The decision maker perceives this orientation with some additional sensory noise, such that the likelihood of the decision maker's observation 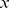 is 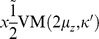 with reduced concentration 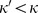, and thus lower precision. Assuming a uniform prior, 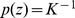, and that the decision maker has learned concentration 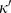 over past trials, her belief over the hidden states follows from Bayes' rule and the definition of the von Mises distribution, and is given by 
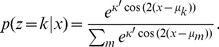
(22)This shows that, independent of the decision maker's decision function 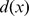, a minimal sufficient statistic of her trial-by-trial belief is the observation, 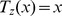, whose dimensionality is always one, independent of the number 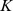 of possible options to choose from.

### Generating Figures 3 and 4

Here we explain how we simulated the diffusion model (DM) and 2-race model to generate Figs. 3 and 4. For both model types, we determined choices and decision times by the decision models, and computed the reaction times by adding a fixed non-decision time of 250 ms to each decision time. All simulations were performed in 1 ms time steps up to a maximum of 2 s, after which the simulation was aborted.

For the DM, we assumed 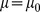 for 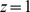 and 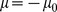 for 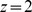, with 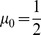. The upper and lower boundaries were time-varying and symmetric around zero, defined by 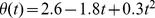 and 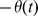 respectively. We have chosen a time-varying boundary to have the belief at the boundary to depend on time. If we had been using a time-invariant boundary instead, this time-dependency of the belief would vanish. Given this setup, we found the decision maker's belief when reaching the upper boundary and thus choosing 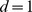 by Eq. (14), resulting in 
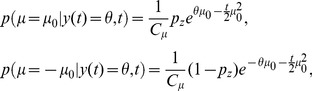
(23)where 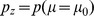, and 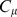 is the normalization constant. We find 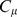 by solving 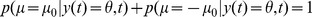, which, when substituted into Eq. (23) a re-arranging the terms, results in the final belief 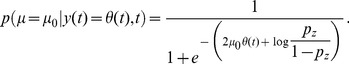
(24)For the uniform prior case in Fig. 3a we generated 10000 trials for each 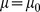 and 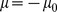, simulating 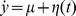 in small time steps until either boundary was reached. We then binned trials by decision time in bins of 250 ms from 250 ms to 1500 ms. To compute performance for each bin we randomly picked 500 trials from this bin in which 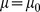, and computed the fraction of times that the upper boundary was reached. Additionally, we plotted the belief for 10 randomly chosen trials from this bin in which this upper boundary was reached.

For the non-uniform prior case in Figs. 3b and 4a we chose 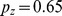. We then generated 10000 trials with 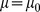 and (to conform to the prior) 5384 trials with 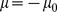 by again simulating 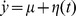 in small time steps. Due to the non-uniform prior, all trajectories reaching the upper boundary caused choice 1, but only trajectories that reached the lower boundary below 
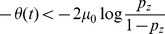
 caused decision 2, and decision 1 otherwise. This strategy arises because the belief at low boundaries is close to 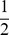. In these cases, the prior might provide more evidence than the likelihood, which might cause a reversal of the decision if prior and likelihood provide evidence for opposing options. Performance and belief were again computed/selected as for the uniform prior case. We computed the estimated belief in Fig. 3b by the fraction of correct choices among 500 trials per bin in which option 1 was chosen.

For the 2-race model in Figs. 3c and 4b we chose 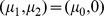 for 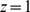 and 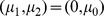 for 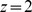, with 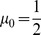. We used the boundary 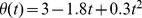, that varied over time but not between races. Given this setup, the decision maker's belief when race 1 is the winning race follows from Eq. (16) and is given by 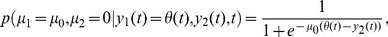
(25)which is a function of both the bound height and the state of the second race. We simulated 10000 trials for each 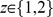 in small time steps, and binned trials by decision time into 250 ms bins from 250 ms to 1750 ms. Performance, trial-by-trial belief, and estimated belief were computed and plotted as for the DM.

### Computing belief in a drift diffusion model with varying difficulty (Figure 5)

We generated Fig. 5 by assuming a decision making diffusion model with diffusion variance 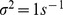, and time-invariant bounds at 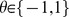. Note that with this choice of diffusion variance, the drift and 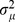 are measured in units of 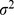. The drift rate was constant within a trial and was chosen across trials to roughly follow 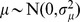 with 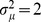 (in units of 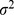). Specifically, we used a point-wise drift rate prior corresponding to a uniform distribution over nine different drift rates, where 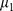 corresponds to the 10^th^ percentile of 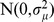, 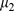 corresponding to the 20^th^ percentile, and so on, up to 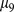 for the 90^th^ percentile (Fig 5a). Reaction times and choice probability were computed analytically using standard results for bounded diffusion models [48]–[49].

For Fig 5b we computed the decision maker's belief under two different assumptions. First, we assumed exact knowledge of the correct point-wise prior, 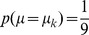 for 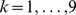, for which the posterior drift rate given that the particle reached the upper or lower bound 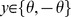 at time 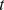 follows Eq. (14), and results in [26]

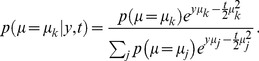
(26)To find the belief we split the prior mass of 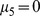 uniformly between 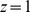 and 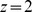 by replacing it by 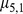 and 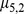 with prior masses 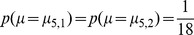, and assign all positive drift rates 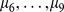 and 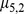 to hidden state 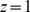, while the remaining drift rates correspond to hidden state 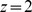. When hitting the upper bound and choosing 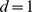, this results in the belief 
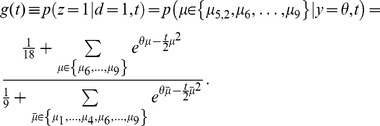
(27)Due to symmetry of prior and task, the same equation holds for the belief of 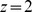 when hitting the lower bound and choosing 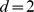. This is the optimal belief the decision maker can hold in this task. Second, we computed the belief based on the assumption that, instead of the correct, point-wise prior, the decision maker assumes a Gaussian zero-mean prior, 
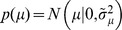
 whose variance 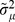 might differ from 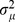. This allowed us to simulate cases in which the decision maker uses an incorrect prior. With this prior, the belief follows again Eqs. (14) and (15), resulting in [26]

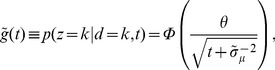
(28)where 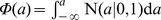 is the standard cumulative Gaussian. To find the average belief per drift rate, as shown in Fig. 5b, we numerically computed the reaction time distribution 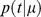 for each 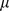 in steps of 1 ms up to 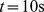 as the solution of a Volterra integral equation of the second kind [47]. Based on this, we computed the average belief for both the point-wise and the Gaussian prior (with 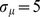) by numerically evaluating the integral 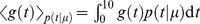. The standard deviation of the belief for the point-wise prior was similarly evaluated by numerical integration based on these reaction-time distributions.

The calibration curves in Fig. 5c were found as follows. Conditional on the absolute drift rate, the probability of performing correct choices is given by 
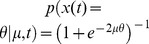
 and is thus independent of the reaction time [48]. In contrast, the belief (Eq. (27)) depends on the reaction time, such that, for a fixed drift rate it will vary across trials even if the probability of choosing the correction option does not. As a result, the calibration curves conditioned on the drift rate, which are given by the function 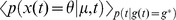 of belief 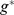, are independent of this belief and thus flat (Fig. 5c). This does not hold anymore as soon as we consider the average calibration curve, as given by 
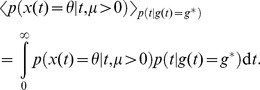
(29)In this case, the probability of making a correct choice depends on the reaction time, as the distribution of these reaction times differs for different drift rates. This becomes particularly clear when expanding the choice probability to give



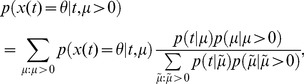
(30)where the fraction inside the sum results from Bayes rule applied to 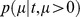. In the above sum, the first term is the known and time-invariant choice probability for a fixed drift rate. The time-dependence is introduced in the second term which is proportional to the probability of reaction time 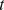 for drift rate 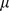 and hence a function of both variables. To evaluate Eq. (29) for a given 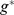 we can utilize the fact that the belief is monotonic in time, such that 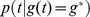 is a Dirac-delta function at the 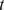 where 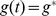. Thus, Eq. (29) results in Eq. (30) evaluated at this time 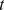, which we find by using the numerical reaction time distributions for fixed drift rates while being again careful about splitting the mass of 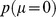 in half. Evaluated for each valid 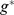 results in the average calibration curve shown in Fig. 5c. In Results (see Eq. (11)) we explain why this average curve follows the identity line.

To simulate the calibration curves for subjects using an incorrect prior 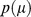, as shown in Fig. 5d, we used the same procedure as to compute the average calibration curve in Fig. 5c. However, rather than using the correct point-wise prior to compute the belief, we assumed the decision maker to utilize a Gaussian prior with different standard deviations. Furthermore, we assumed the actual drift rates to follow a Gaussian prior such that in Eq. (30) the sum turns into an integral that we solved numerically. Specifically, to simulate a too-wide prior, we assumed the actual prior to be a zero-mean Gaussian with standard deviation 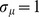, while the decision maker assumed 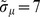. For the too narrow prior we used the actual prior width 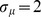 while setting the assumed prior width to 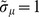.

### Modeling miscalibrations by the decision variable partition model

The decision variable partition model [32]–[33] is a popular model based on signal detection theory to explain various types of miscalibration. In this model, the decision maker observes two random variables, 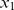 and 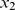 (for example, two weights that need to be compared), where one is drawn from the “correct”, and the other from the “incorrect” distribution and the aim of the decision maker is to identify the one associated with the correct distribution. We formalize this by 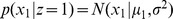 and 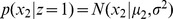 for 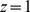, and 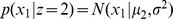 and 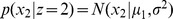 with flipped means for 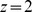. Then, it is easy to show that the hidden state posterior upon observing 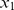 and 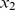 is given by 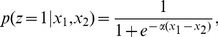
(31)where 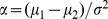 determines the difficulty of the task. Thus, the decision rule that maximizes this posterior is to choose 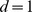 if 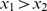 and 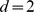 otherwise. This leads to the optimal belief 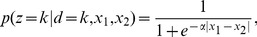
(32)which is a monotonically increasing function of 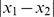 (a larger difference in perceived weights increases the confidence). Instead of using the optimal belief according to Eq. (32), the decision variable partition model proposes to partitioning the space of 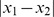 into arbitrarily chosen bins and to assign each of these bins a different confidence rating. This way it is able to capture any deviation from perfect calibration, as long as the decision maker's performance grows monotonically with belief. Furthermore, it captures the hard-easy effect by leaving the partitioning unchanged for different task difficulties while the optimal belief would require adjusting 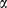 in Eq. (32) accordingly.
